# Selective enhancement of glutamate-mediated pressor responses after GABA_A_ receptor blockade in the RVLM of sedentary versus spontaneous wheel running rats

**DOI:** 10.3389/fphys.2012.00447

**Published:** 2012-11-26

**Authors:** Patrick J. Mueller, Nicholas A. Mischel

**Affiliations:** Department of Physiology, Wayne State University School of MedicineDetroit, MI, USA

**Keywords:** blood pressure, exercise, brainstem, glutamate, GABA

## Abstract

Overactivity of the sympathetic nervous system (SNS) is a hallmark of many cardiovascular diseases. It is also well-known that physical inactivity independently contributes to cardiovascular diseases, likely in part via increased SNS activity. Recent work from our laboratory has demonstrated increased SNS responses in sedentary animals following either direct activation or disinhibition of the rostral ventrolateral medulla (RVLM), an integral cardiovascular brainstem region. These data led us to hypothesize that the interaction between excitation and inhibition of the RVLM is altered in sedentary versus physically active animals. To test this hypothesis, we recorded mean arterial pressure (MAP) and lumbar sympathetic nerve activity (LSNA) in Inactin anesthetized rats that were housed for 8–12 weeks with or without access to a running wheel. Pressor responses to direct activation of the RVLM with glutamate were similar between groups under intact conditions. However, blockade of γ-aminobutyric acid (GABA)_A_ receptors with bicuculline selectively enhanced pressor responses to glutamate in sedentary animals. Interestingly, LSNA responses to glutamate were not enhanced in sedentary versus active animals in the presence or absence of tonic GABAergic tone. These results suggest that sedentary compared to active conditions enhance GABAergic inhibition of glutamate-sensitive neurons in the RVLM that are involved in blood pressure regulation, and by mechanisms that do not involve LSNA. We also speculate that regular physical activity has differential effects on SNS activity to specific vascular beds and may reduce the risk of developing cardiovascular diseases via changes occurring in the RVLM.

## Introduction

There is now convincing evidence that cardiovascular diseases including hypertension and heart failure are associated with overactivity of the sympathetic nervous system (SNS) (Zucker et al., 2001; Esler et al., 2003; Fisher et al., 2009; Malpas, 2010). Since one of the primary risk factors for cardiovascular disease is a lack of regular exercise (i.e., a sedentary lifestyle), it is not surprising that studies have also demonstrated evidence of sympathetic overactivity in sedentary versus physically active individuals (Meredith et al., 1991; Mueller, 2010). In particular, we and others have proposed that changes in sympathetic regulation in sedentary versus physically active animals are due to alterations in the rostral ventrolateral medulla (RVLM), an important brainstem region involved in sympathetic outflow (Becker et al., 2005; Martins-Pinge et al., 2005; Mueller, 2010). Indeed, RVLM neurons receive a variety of cardiovascular and exercise-related inputs (Dampney, 1994a,b; Guyenet and Stornetta, 2004). Ionotropic glutamate receptors in the RVLM appear to mediate pressor response to activation of skeletal muscle afferents by contraction or direct stimulation (Bauer et al., 1989; Kiely and Gordon, 1993; Ally, 1998). Despite the evidence in favor of a role for the RVLM in the acute response to exercise, the mechanisms by which chronic exercise (or lack thereof) alters control of SNS activity at the level of the RVLM remain to be fully elucidated.

The activity of RVLM neurons is regulated primarily by the excitatory neurotransmitter glutamate and by the inhibitory neurotransmitter γ-amino butyric acid (GABA), with contributions from other neurotransmitters as well (Dampney, 1994a; Guyenet and Stornetta, 2004; Pilowsky et al., 2008). Recent data from our laboratory and others have suggested that sedentary versus physically active conditions produce a relative enhancement in the responsiveness of RVLM neurons to direct excitation by glutamate but not all neurotransmitters (Becker et al., 2005; Martins-Pinge et al., 2005; Mueller, 2007). In addition, alterations in GABAergic neurotransmission at the level of the RVLM have been proposed to contribute to changes in sympathoexcitation in a variety of animal models (Adams et al., 2007; Huber and Schreihofer, 2011), including models of physical activity and inactivity (Moffitt et al., 2002; Mueller, 2007). Although under acute experimental conditions, glutamate and GABA can provide parallel excitation and inhibition, respectively in the RVLM (Miyawaki et al., 1996), there are no studies to our knowledge that have examined this interaction in a chronic model of physical (in)activity.

The primary purpose of this study was to examine the influence of sedentary versus physically active conditions on the interaction between glutamate-mediated excitation and GABAergic-mediated inhibition at the level of the RVLM. Similar to our previous studies (Mueller, 2007; Mischel and Mueller, 2011), we hypothesized that sedentary versus physically conditions would result in enhanced pressor and sympathoexcitatory responses to direct excitation of the RVLM and that these responses would be further augmented by blockade of tonic GABAergic transmission. To test these hypotheses, we recorded blood pressure, heart rate (HR), and lumbar sympathetic nerve activity (LSNA) responses to RVLM microinjections in sedentary and physically active rats.

## Materials and methods

### Drugs

Inactin, L-glutamate, and bicuculline methiodide were obtained from Sigma Chemical (St. Louis, MO). Drugs used for microinjection were dissolved in artificial cerebrospinal fluid (aCSF) in which pH had been adjusted to 7.3–7.5 using sodium hydroxide or hydrochloric acid.

All surgical and experimental procedures were approved by the Institutional Animal Care and Use Committee of Wayne State University and conducted in accordance with the American Physiological Society's “Guiding Principles in the Care and Use of Animals.” All animals received food (Purina Lab Diet #5001, Purina Mills, Richmond, IN) and tap water *ad libitum*.

### Daily spontaneous running

Forty-nine male Sprague–Dawley rats (initially 75–100 g, Harlan, Indianapolis, IN) were used for these studies. Animals were housed individually in cages with (physically active group, *n* = 24) or without running wheels (sedentary group, *n* = 25) for 8–12 weeks. Running wheels were purchased from a national vendor (Tecniplast, Eaton, PA; wheel diameter 34 cm diameter). Running distances were measured via bicycle computers (Sigma Sport, Olney, IL) calibrated to the diameter of the running wheel and recorded daily by laboratory personnel. Previous studies have demonstrated that rats allowed to run spontaneously on running wheels exhibit increases in maximal oxygen consumption, increases in heart weight-to-body weight ratio, and other exercise-related adaptations (Nelson et al., 2010).

### Surgical procedures

Following 8–12 weeks of sedentary or physically active conditions, animals were instrumented acutely for brainstem microinjections while recording mean arterial pressure (MAP), HR, and LSNA (Mueller, 2007; Mischel and Mueller, 2011). Briefly, animals were anesthetized with isoflurane (2% in 100% O2) so that arterial and venous femoral catheters could be implanted to measure arterial pressure and for the administration of drugs, respectively. Following a midline abdominal incision, the abdominal aorta was gently retracted and a section of the lumbar chain was isolated caudal to the renal vein. The nerve was carefully placed on silver wire electrodes before encasing in polyvinylsiloxane gel (Darby Dental Supply Co., Inc., Westbury, NY). The abdominal incision was sutured around the electrode as it exited the peritoneal cavity. A ground wire was attached to the incision to reduce noise. A tracheostomy was performed and the animals were ventilated artificially with a mixture of isoflurane and oxygen (2% in 100% O2) via an endotracheal tube. Rats were placed in a prone position in a stereotaxic device (Kopf, Tujunga, CA) and following a midline incision at the level of the occipital bone, the brainstem was exposed by retracting the underlying muscles, performing a partial occipital craniotomy, and removing the atlanto-occipital membrane.

Once all surgical procedures were complete, an initial infusion of Inactin (thiobutabarbital sodium, 0.025 ml/min, 100 mg/kg, i.v.) was performed over 20–30 min during which the isoflurane was slowly reduced. Following the infusion, Inactin was given in supplemental doses (5 mg, i.v.) until an appropriate level of anesthesia was maintained in the absence of isoflurane. Anesthesia was deemed appropriate by lack of a withdrawal reflex to firm pinch. Animals continued to be ventilated (60–80 breaths/min) with a mixture of 100% O_2_ and room air. Arterial blood gases were determined and were maintained in the physiological range (*P*_O_2__ > 100 mmHg, *P*_CO_2__ between 35 and 40 mmHg) by adjusting the rate or volume of the ventilator. Body temperature was maintained near 37°C via the use of a heating pad and measured throughout the experiment with a rectal thermometer. In order to diminish outside electrical noise, experiments were performed inside a Faraday cage.

### Microinjections

We performed microinjections based on previously published techniques in our laboratory (Mueller and Hasser, 2006; Mueller, 2007). Briefly, once the animal was in the stereotaxic frame, the head was positioned at a downward angle such that calamus scriptorius was 2.4 mm caudal to the interaural line and the brainstem oriented in the horizontal plane (Kiely and Gordon, 1994; Moffitt et al., 2002). Triple-barrel glass micropipettes were drawn by a pipette puller to an outside tip diameter of 30–60 μm, filled with appropriate drugs, and inserted into the dorsal surface of the brainstem with the use of a surgical dissecting microscope. The RVLM was located with the following coordinates using calamus scriptorius as a reference point: 0.9–1.1 mm rostral and 1.7–2.2 mm lateral to calamus scriptorius, and 3.5–3.7 mm ventral to the dorsal surface of the medulla. Micropipettes were connected via polyethylene tubing to a commercially available picoejection system (Toohey Company, Fairfield, NJ). The volume of microinjections was monitored directly by visualizing the meniscus in each barrel with the aid of a compound microscope (150×) that contained a calibrated reticule. The RVLM was functionally identified by observing pressor and sympathoexcitatory responses to a standard dose and volume of glutamate (10 mM, 30 nl). Microinjection sites were marked with 2% Chicago sky blue dye (30 nl). At the end of the experiments, animals were overdosed with Fatal-Plus euthanasia solution (Vortech, Dearborn, MI, 0.2 ml) and brains were removed and placed in 4% phosphate buffered formalin solution. Following post fixation, brains were transferred to 30% sucrose for a minimum 48-h infiltration. The hindbrain was frozen and cut into 30 μm sections on a cryostat. Coronal sections were mounted on gel-coated slides and a bright-field microscope was used to determine the center of the dye spot and its location in the brainstem with the aid of a rat brain atlas (Paxinos and Watson, 2007). The dye spot location was represented graphically on a modified diagram from the rat atlas (Paxinos and Watson, 2007).

### Protocol #1—effect of increasing concentrations of glutamate on responses to excitation of the RVLM in sedentary and physically active rats

We tested the hypothesis that responses to excitation of the RVLM were enhanced in sedentary animals compared to animals that ran spontaneously on running wheels. Unilateral microinjections of specific concentrations of glutamate (1, 10, and 100 mM) were performed at a constant volume of 30 nl (30, 300, and 3000 pmol total, respectively). Specific concentrations were ejected from individual barrels of the triple-barrel micropipette, performed in a random order, and a minimum of 5 min of recovery time was allowed between responses. Control injections of vehicle (i.e., aCSF) produced little or no response in MAP, HR, and LSNA (see Results).

### Protocol #2—effect of GABA_A_ receptor blockade on responses to glutamate

We hypothesized that responses to glutamate would be enhanced in sedentary rats in the absence of tonic GABAergic tone compared to animals that ran spontaneously on running wheels. To test this hypothesis, GABA receptors were blocked unilaterally with the GABA_A_ receptor antagonist, bicuculline (5 mM, 60 nl, or 300 pmol total) prior to subsequent injections of glutamate. Responses to glutamate were compared before and 5, 15, 30, and 45 min after bicuculline. The concentration of bicuculline used was based on previous studies in which bicuculline was shown to inhibit GABA_A_ receptor activation in the RVLM (Miyawaki et al., 2002; Moffitt et al., 2002; Horiuchi et al., 2004) or block arterial baroreflex-mediated changes in sympathetic nerve activity (Sun and Guyenet, 1985; Blessing, 1988; Dampney et al., 1988; Guyenet and Stornetta, 2004; Mueller, 2007).

### Protocol #3—effect of control injections on responses to glutamate

Microinjections of glutamate were tested in the presence and absence of aCSF (60 nl) to determine the influence of vehicle, volume, or time on responses to repeat microinjections of glutamate. The time course and size of injections were similar to those used for bicuculline in Protocol #2.

### Data collection and analysis

A computer data acquisition system (Power Lab, ADInstruments, Colorado Springs, CO) was used to collect all experimental data. Raw LSNA was monitored on a Tektronix oscilloscope and a Grass preamplifier (P511) was used to amplify (20,000×) and filter LSNA (3 kHz low pass and 30 Hz high pass filter). LSNA was electronically rectified, integrated, and averaged using a time constant of 28 ms. Changes in LSNA were calculated as a percentage of control prior to each microinjection. The average amplified voltage (20,000×) was also compared between all animals as an estimate of baseline sympathetic nerve activity. Hexamethonium (30 mg/kg) and atropine methyl bromide (1 mg/kg, i.v.) were administered as ganglionic blocking agents to determine background noise. Background noise was subtracted from each animal and the remaining signal was defined as LSNA.

### Statistical analysis

Baseline hemodynamic variables, body weights, and organ weights were analyzed by Student's *t*-test. MAP, HR, or LSNA changes to specific concentrations of glutamate were analyzed by Two-Way analysis of variance (ANOVA) with repeated measures. When ANOVA indicated a significant interaction, differences between individual means were assessed by *post-hoc* Holm-Sidak test according to a commercially available software package (SigmaStat 3.0, SPSS Inc., Chicago, IL). In one instance square root transformations were performed on MAP/Glutamate dose response data to achieve normality before Two-Way ANOVA. The transformation did not change the statistical outcome of the test (*p* > 0.05).

For protocols 2 and 3 (bicuculline and aCSF, respectively), data were combined initially and subjected to multivariate and repeated measures analyses using the General Linear Model (GLM) program in IBM SPSS for Windows (Version 19.0 Armonk, NY: IBM Corp). Based on both theory and the literature regarding the time course of bicuculline effects, our expectation was that any significant effects over the time points measured in both protocols (control, 5′, 15′, 30′) would be represented by the quadratic trend component (i.e., the two middle values would be higher than the first and fourth). Although preliminary analyses using both the multivariate and the repeated measures options gave similar results, the quadratic effects accounted for almost all of the between time variance. In the repeated measures analysis for the quadratic scores, we found both a significant time by experiment interaction and a time by group interaction (*p* < 0.05 for each). The former interaction justified testing the individual protocols (Bicuculline and aCSF) separately. We then performed simple main effects *t*-tests on the quadratic scores. As expected and borne out by the analysis, all of the effects revealed by ANOVA were due to bicuculline. As follow up to the significant group by quadratic time interaction (for bicuculline only), we analyzed the simple main effects of group at each time point using Holm–Sidak *post-hoc* tests provided by the Two-Way RM ANOVA. The results of these analyses are provided in the graphs and justify separation of the two protocols graphically.

For all analyses, a probability of *p* < 0.05 was considered statistically significant and *p*-values less than or equal to 0.1 for main effects are reported for clarity (Curran-Everett and Benos, 2004). Data are expressed as mean ± SEM.

## Results

### Sedentary versus physically conditions

Table 1 contains baseline characteristics in sedentary versus physically active animals. Physically active animals ran for an average of 10.4 ± 0.3 weeks which resulted in total distances ran of over 200 km (Table 1). Sedentary animals remained in their cages for similar periods of time without access to running wheels. Body weight was higher in sedentary animals (*p* < 0.05); however, there were no significant differences in baseline MAP, HR, or the average amplified LSNA voltage between groups. Similarly, baseline MAP, HR, and average amplified LSNA voltage was not significantly different between groups prior to each protocol (Table 2).

**Table 1:** **Baseline characteristics of sedentary versus physically active animals**.

	**Body weight (g)**	**MAP (mmHg)**	**HR (bpm)**	**LSNA (mV.s)**	**Total running distance (km)**
Sedentary (*n* = 25)	407 ± 7^*^	102 ± 2	279 ± 4	1.22 ± 0.11	–
Physically active (*n* = 24)	385 ± 9	105 ± 2	284 ± 4	1.18 ± 0.17	220 ± 24

MAP, mean arterial pressure; HR, heart rate; LSNA, average amplified lumbar sympathetic nerve voltage;

* p < 0.05 compared to physically active animals.

**Table 2:** **Baseline values for individual protocols**.

	**MAP (mmHg)**	**HR (bpm)**	**LSNA (mV.s)**
**GLUTAMATE DOSE-RESPONSE**			
Sedentary (*n* = 17)	103 ± 2	276 ± 5	1.08 ± 0.11
Physically active (*n* = 16)	104 ± 2	278 ± 5	1.30 ± 0.25
**GLUTAMATE ± BICUCULLINE**			
Sedentary (*n* = 10)	106 ± 2	294 ± 6	1.61 ± 0.17
Physically active (*n* = 10)	105 ± 2	289 ± 8	1.18 ± 0.21
**GLUTAMATE ± ACSF**			
Sedentary (*n* = 8)	107 ± 3	299 ± 7	1.73 ± 0.28
Physically active (*n* = 7)	97 ± 6	280 ± 6	1.65 ± 0.39

MAP, mean arterial pressure; HR, heart rate; LSNA, average amplified lumbar sympathetic nerve voltage; ACSF, artificial cerebrospinal fluid. There were no significant differences in any baseline variable between groups for any of the protocols.

### Protocol #1—effect of increasing concentrations of glutamate in the RVLM of sedentary versus physically active rats

**Figure 1:**
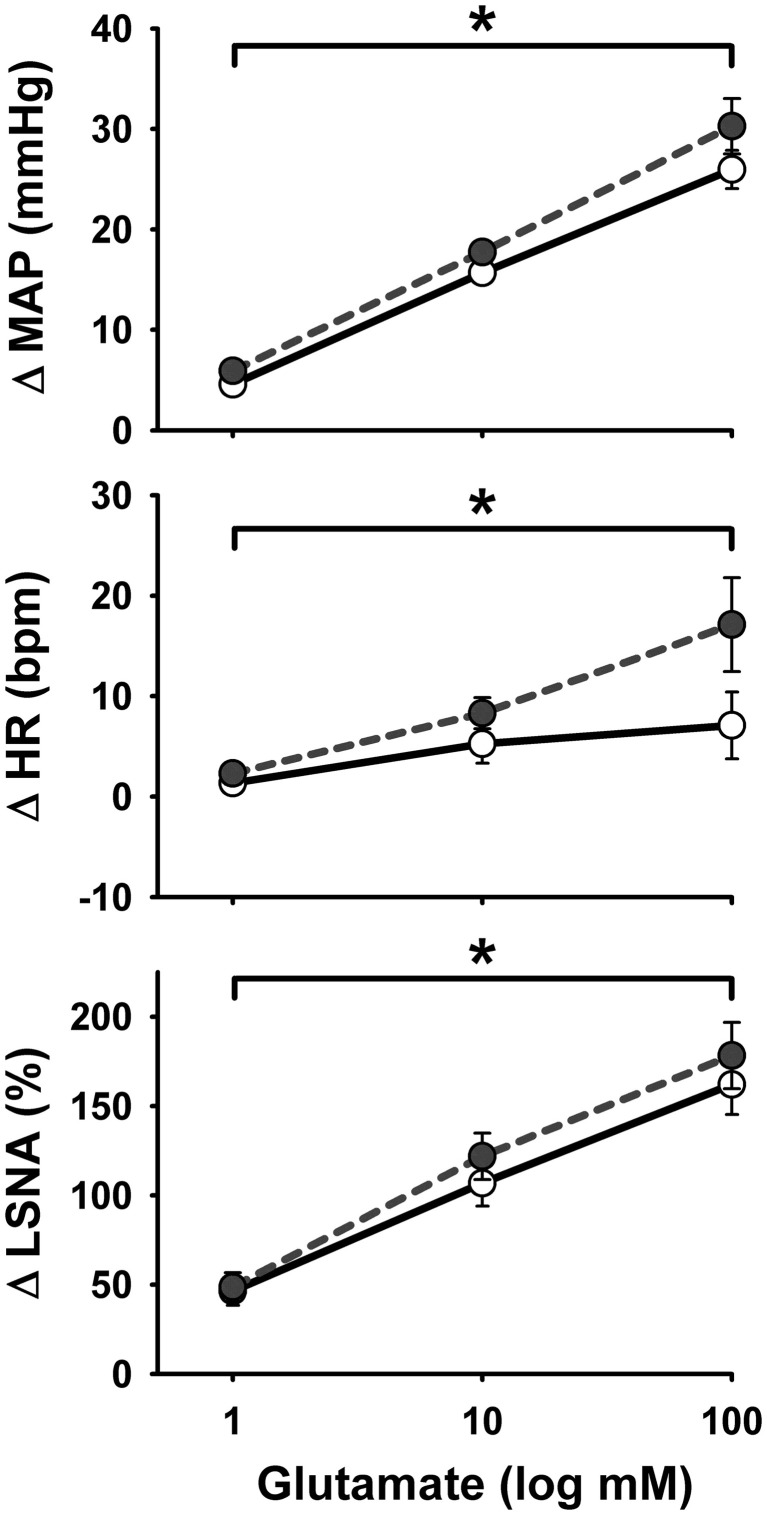
**Peak mean arterial pressure (MAP), heart rate (HR), and lumbar sympathetic nerve activity (LSNA) responses to activation of the rostral ventrolateral medulla with increasing concentrations of glutamate (30 nl) in sedentary (filled circles, *n* = 16) and physically active rats (open circles, *n* = 17).** There was a significant effect of glutamate concentration to increase MAP, HR, and LSNA (^*^p < 0.05) but responses did not differ between groups.

**Figure 2:**
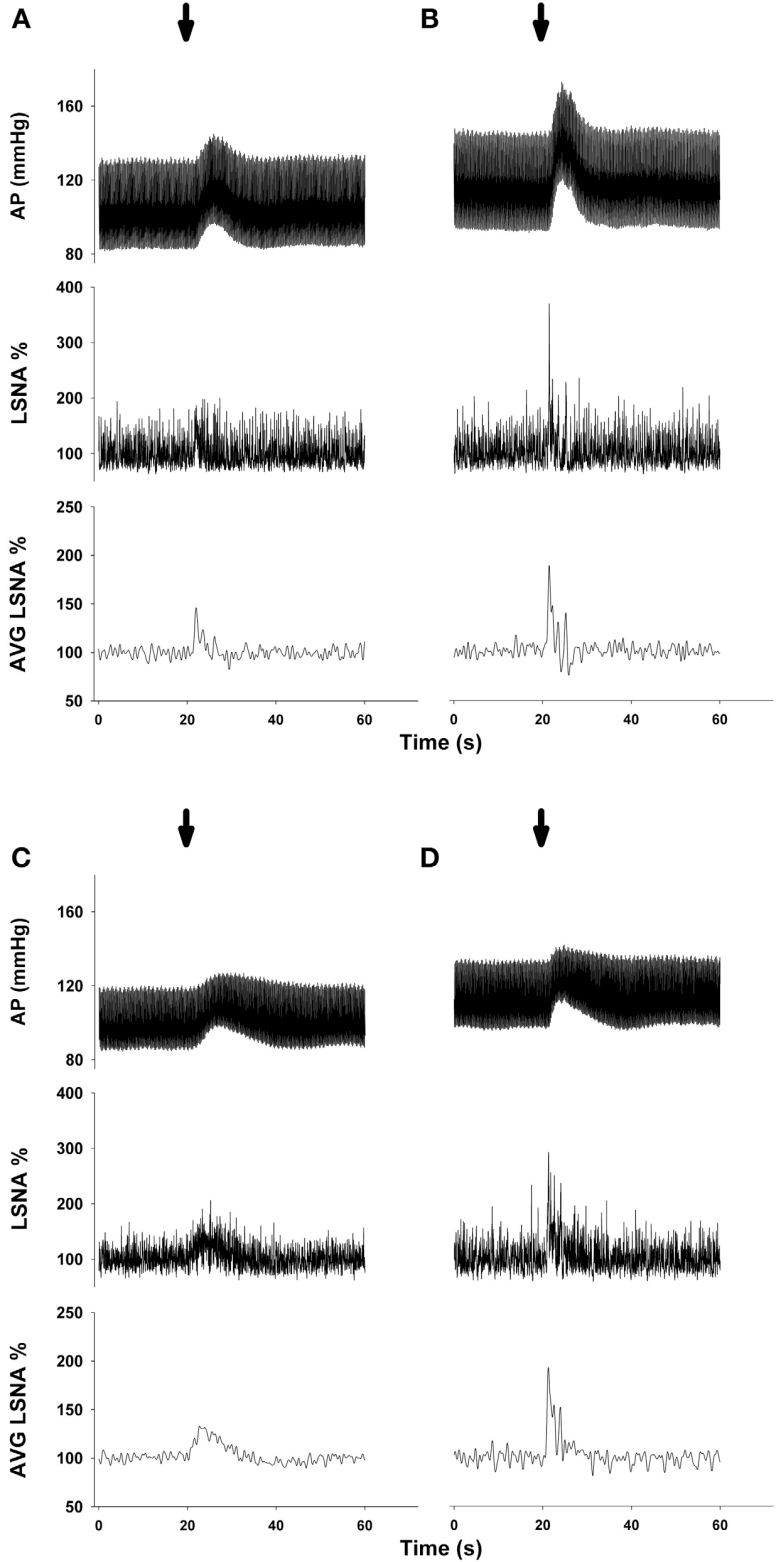
**Representative examples of arterial pressure (AP) and LSNA responses to unilateral microinjections of glutamate (30 nl, 10 mM) into the rostral ventrolateral medulla before and after microinjection of the GABA_A_ antagonist bicuculline (60 nl, 5 mM). (A)** Control response to glutamate in one sedentary rat. **(B)** Response to glutamate in the same sedentary rat 5 min after bicuculline. **(C)** Control response to glutamate in one physically active rat. **(D)** Response to glutamate in the same physically active rat 5 min after bicuculline. Note enhanced pressor response only in sedentary rat after bicuculline and enhanced LSNA response in both sedentary and physically active rats following bicuculline. Arrow represents timing of glutamate microinjection. Abbreviations are as defined in Figure 1.

Representative MAP and LSNA responses in one sedentary and one physically active rat to RVLM microinjection of glutamate (30 nl, 10 mM) are shown in Figures 2A,C. Averaged peak changes in MAP, HR, and LSNA to microinjections of glutamate (30 nl, 1–100 mM) are shown in Figure 1. As in our previous studies (Mueller, 2007; Mischel and Mueller, 2011), glutamate produced concentration-dependent increases in MAP, HR, and LSNA (*p* < 0.001, main effect of dose for all three variables). The pressor responses to increasing concentrations of glutamate were not statistically different between sedentary and physically active animals (*p* = 0.10 for main effect) and the lack of a significant interaction did not allow for testing for differences at individual concentrations between groups (*p* = 0.872). HR responses to glutamate microinjections into the RVLM were small in general (<20 bpm) and there was neither a main effect of sedentary condition (*p* = 0.215) nor a significant interaction (*p* = 0.489) that would allow for testing differences at individual doses. Lastly, unlike our previous studies in which we demonstrated enhanced sympathoexcitation in splanchnic (Mischel and Mueller, 2011) or lumbar sympathetic nerves (Mueller, 2007) of sedentary animals, increases in LSNA were not significantly different between sedentary and physically active animals (*p* = 0.576 for main effect; *p* = 0.646 for interaction).

### Protocol #2—effect of GABA_A_ receptor blockade on responses to glutamate

**Figure 3:**
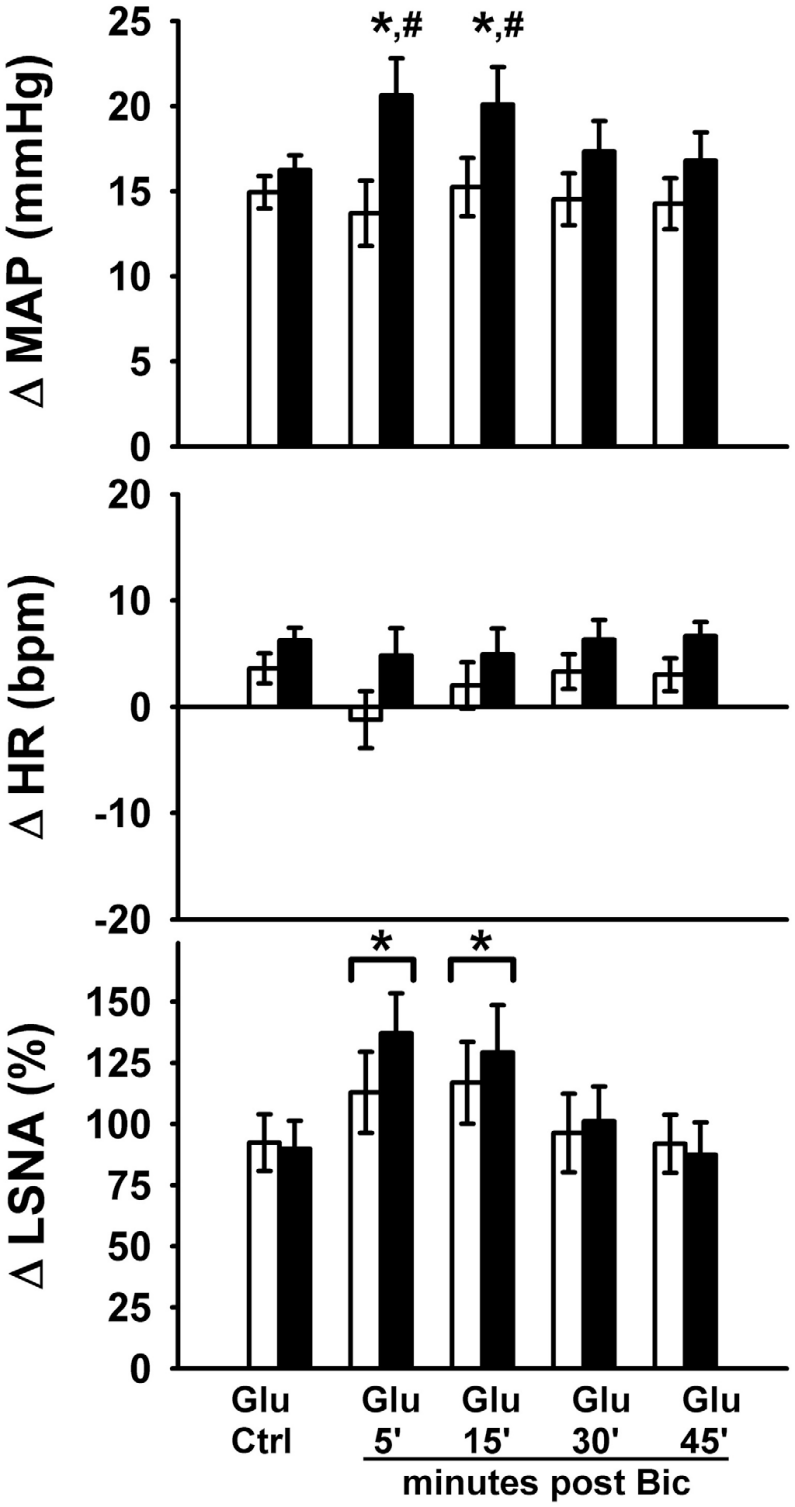
**Peak MAP, HR, and LSNA responses to activation of the rostral ventrolateral medulla with glutamate (10 mM, 30 nl) in the presence or absence of GABA_A_ receptor blockade with bicuculline (5 mM, 60 nl).** Sedentary (filled bars, *n* = 10) and physically active rats (open bars, *n* = 10) had similar pressor and sympathoexcitatory response to glutamate prior to bicuculline (Glu Ctrl). Following bicuculline, sedentary animals exhibited a selective enhancement in pressor responses to glutamate at 5 and 15 min (^*^p < 0.05), resulting in significantly greater pressor responses compared to physically active animals (^#^*p* < 0.05 at 5 and 15 min). LSNA response were similarly enhanced in both groups at 5 and 15 min (^*^with bracket, *p* < 0.05). Responses in both groups returned to control within 30 min. The small HR responses were also significantly different between groups (*p* < 0.05). Abbreviations are as defined in Figure 1.

Representative MAP and LSNA responses in one sedentary and one physically active rat to RVLM microinjection of glutamate (30 nl, 10 mM) before and after bicuculline are shown in Figure 2. Averaged peak changes in MAP, HR, and LSNA to microinjections of glutamate (30 nl, 10 mM) with and without bicuculline are shown in Figure 3. As in Protocol #1, control responses to glutamate (30 nl, 10 mM) produced increases in MAP and LSNA and small changes in HR (<10 bpm) in both sedentary and physically active animals. Unilateral injections of bicuculline increased baseline MAP (Sedentary Δ9 ± 2 mmHg; Physically Active Δ13 ± 2 mmHg), HR (Sedentary –Δ7 ± 6 bpm; Physically Active Δ5 ± 2 bpm), and LSNA (Sedentary Δ 3 ± 4%; Physically Active Δ9 ± 3%) prior to the subsequent injection of glutamate at 5 min. These changes were not significantly different between groups (*p* > 0.05). Similar to control injections of glutamate, microinjection of glutamate 5 min following bicuculline also produced increases in MAP, HR, and LSNA. However, increases in MAP produced by glutamate injections at both 5 and 15 min after bicuculline were selectively enhanced in sedentary animals such that responses were significantly greater in sedentary versus physically active animals (*p* < 0.05 for main effects, interaction, and *post-hoc* tests). Glutamate-induced pressor responses at 30 and 45 min were similar to glutamate control responses suggesting recovery of responses and consistent with the time course of action of bicuculline in producing disinhibition of the RVLM (Miyawaki et al., 2002; Mueller, 2007). Interestingly, glutamate-induced increases in LSNA were also enhanced by bicuculline at 5 and 15 min in both groups (*p* < 0.05 for main effect and multiple comparisons within main effect) but were not significantly different between sedentary and physically active animals (*p* > 0.05 for main effect). At 30 and 45 min after bicuculline, increases in LSNA produced by glutamate were not significantly different than control glutamate responses (*p* > 0.05 for multiple comparisons within main effect) and were not different between groups, also suggesting full recovery of responses from bicuculline in a manner consistent with its time course of action. Lastly, bicuculline produced a slight but significant main effect on the small HR responses (*p* < 0.05 for main effect), but there was neither a main effect of sedentary condition nor interaction to allow testing for further differences (*p* > 0.05 for both).

### Protocol #3—effect of control injections on responses to glutamate (Figure 4)

To control for potential volume-, vehicle-, or time-related effects of bicuculline injections on responses to glutamate microinjections, we performed a series of experiments utilizing microinjections of the bicuculline vehicle (i.e., aCSF) before and after repeat microinjections of glutamate into the RVLM. As in Protocols 1 and 2, control responses to glutamate (30 nl, 10 mM) produced increases in MAP and LSNA and small changes in HR (<10 bpm) which were not different between sedentary and physically active animals. Similar to previous studies (Mueller, 2007), microinjections of aCSF (60 nl) had little or no effect on baseline MAP (Sedentary –Δ2 ± 2 mmHg; Physically Active Δ0 ± 1 mmHg), HR (Sedentary –Δ1 ± 3 bpm; Physically Active –Δ1 ± 1 bpm), or LSNA (Sedentary Δ1 ± 3%; Physically Active Δ0 ± 1%). Responses to repetitive microinjections of glutamate at 5, 15, and 30 min were also not significantly different than control glutamate microinjections for increases in MAP and LSNA, and small changes in HR (*p* > 0.05 for main effect of aCSF). Lastly, responses to repetitive microinjections were not significantly different between groups (*p* > 0.05 for main effect of physically active condition).

**Figure 4:**
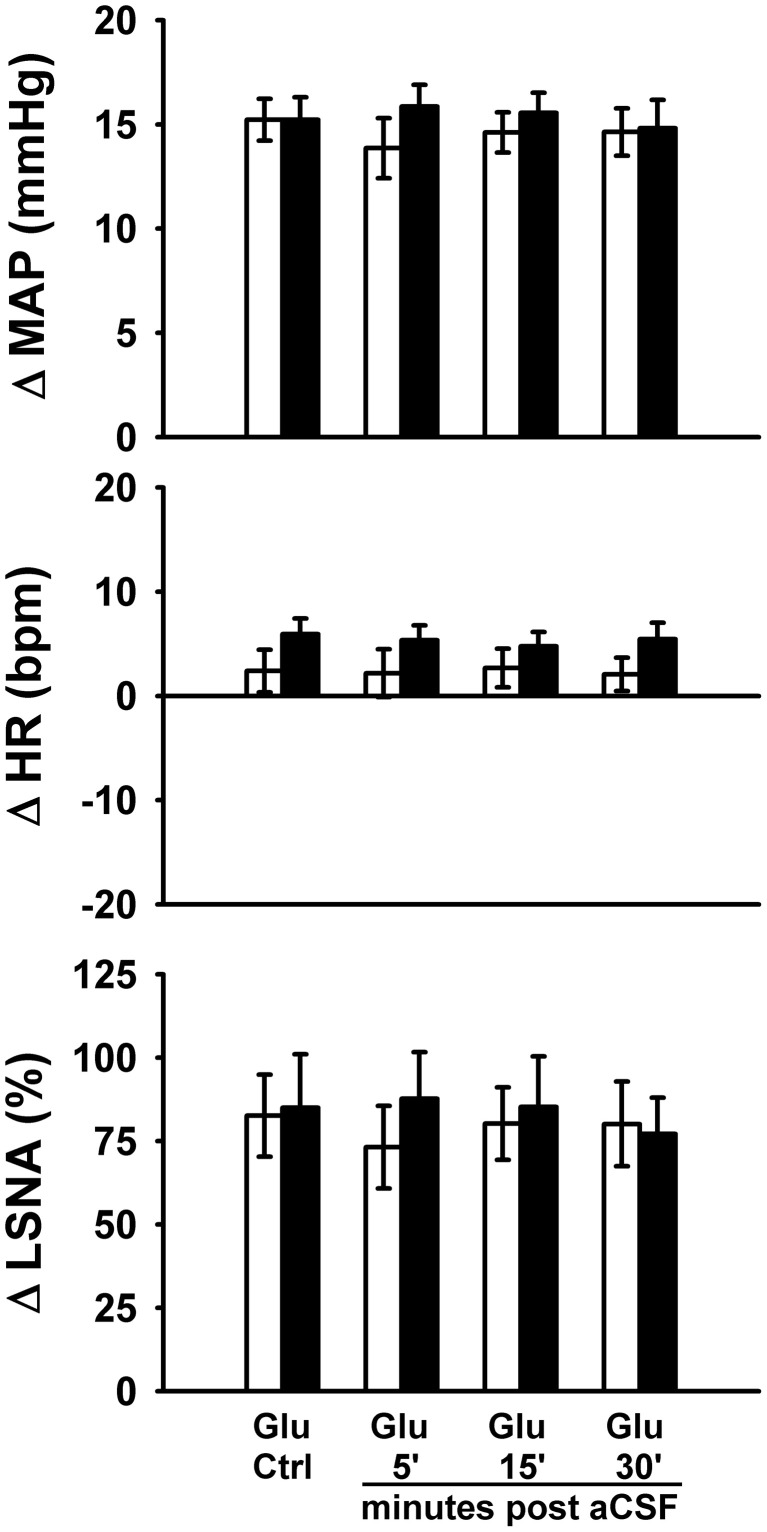
**Peak MAP, HR, and LSNA responses to activation of the rostral ventrolateral medulla with glutamate (10 mM, 30 nl) in the presence or absence of artificial cerebrospinal fluid injections (aCSF, 60 nl).** aCSF had no significant effect on responses in either sedentary (filled bars, *n* = 8) or physically active rats (open bars, *n* = 7) over the entire time course of injections and there were no significant differences between groups. Abbreviations are as defined in Figure 1.

### Histology (Figure 5)

Microinjection sites marked with 2% Chicago sky blue dye (30 nl) were recovered in 16 sedentary and 17 physically active animals used in these studies. As in our previous studies (Mischel and Mueller, 2011), all microinjections sites were near the caudal pole of the facial nucleus and located ventral to the nucleus ambiguus, lateral to the pyramidal tract, and medial to the spinal trigeminal tract, which characterize the boundaries of the RVLM established by previous studies (Guyenet, 2006; Schreihofer and Sved, 2011).

**Figure 5:**
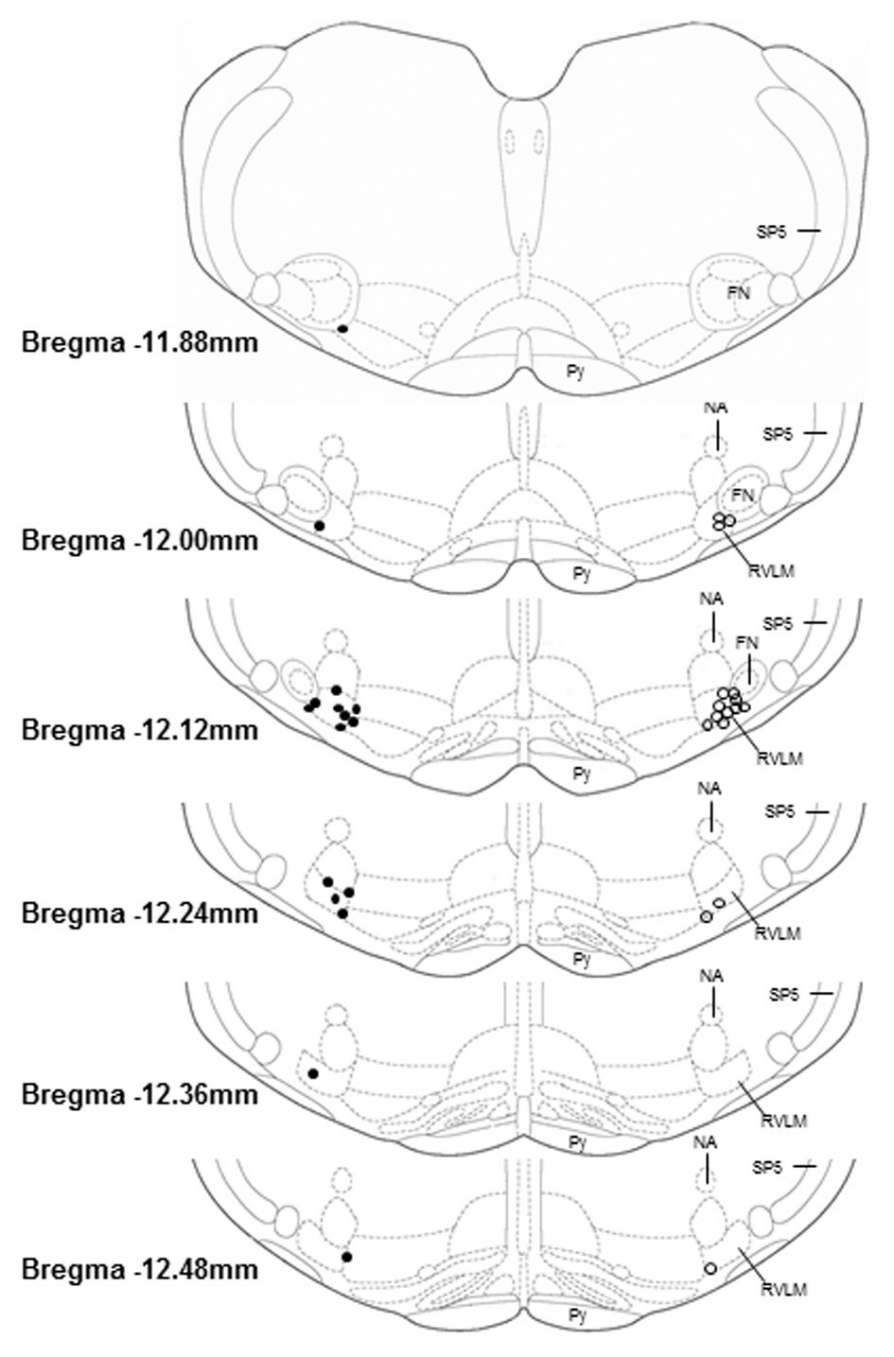
**Histological representation of microinjection sites plotted on brainstem sections that have been modified from a standard rat atlas (Paxinos and Watson, 2007).** Unilateral injection sites from sedentary (filled circles, *n* = 16) and physically active animals (open circles, *n* = 16) are plotted on opposite sides for clarity. All microinjection sites were associated with pressor and sympathoexcitatory responses to glutamate; were confined within the general boundaries of the rostral ventrolateral medulla (RVLM); and were distributed similarly between groups. FN, facial nucleus; NA, nucleus ambiguus; Py, pyramidal tract; SP5, spinal trigeminal tract.

## Discussion

The primary purpose of this study was to examine the influence of sedentary versus physically active conditions on the interaction between glutamate-mediated excitation and GABAergic-mediated inhibition at the level of the RVLM. We hypothesized that sedentary versus physically conditions would result in enhanced pressor and sympathoexcitatory responses to direct excitation of the RVLM and that these responses would be further augmented by removal of tonic GABAergic transmission. Our hypothesis was confirmed in part by the selective enhancement in the pressor responses to glutamate in sedentary animals following GABA receptor blockade in the RVLM. In contrast, and contrary to our hypothesis, lumbar sympathetic nerve responses to direct excitation of the RVLM were similar in both groups under intact conditions or following GABA_A_ receptor blockade. Collectively, these data provide several important new findings that increase our understanding of alterations in neural control of the circulation between sedentary and physically active animals. (1) Sedentary versus physically active conditions result in enhanced GABAergic modulation of glutamate-sensitive neurons in the RVLM that are involved in blood pressure regulation. (2) Sedentary versus physically active conditions appear to enhance the excitability of glutamate-sensitive neurons in the RVLM in the absence of GABAergic modulation. (3) Enhanced pressor responses observed in sedentary animals following GABA_A_ receptor blockade do not appear to be mediated by lumbar sympathetic nerves. We speculate that sedentary versus physically active conditions produce alterations in RVLM neurons which lead to differential changes in regulation of blood pressure and may contribute to the increased prevalence of cardiovascular disease in sedentary individuals.

One of the most important and novel observations in our study was the enhanced pressor response to activation of the RVLM following blockade of GABA_A_ receptors in sedentary but not physically active animals. These data suggest two important concepts regarding the influence of sedentary versus physically active conditions on the regulation of blood pressure at the level of the RVLM. First, GABA appears to modulate glutamate-mediated increases in arterial pressure produced at the level of the RVLM differently in sedentary versus physically active animals. Second, in the absence of modulation by GABA_A_ receptors, RVLM neurons involved in blood pressure regulation appear to be more sensitive to glutamatergic excitation in sedentary animals. Enhanced GABAergic modulation in sedentary animals appears to restrain excessive blood pressure responses from occurring under intact conditions since responses were similar in physically active animals. However, when GABAergic inhibition is removed (via receptor blockade in this study), the enhanced pressor response to glutamate is allowed to be fully expressed resulting in significantly larger pressor responses compared to physically active animals. Consistent with this finding are previous studies in which enhanced sympathoexcitation has been observed in sedentary animals under conditions in which GABAergic transmission would be expected to be reduced. For example, enhanced sympathoexcitation occurs in sedentary animals during baroreceptor unloading (DiCarlo and Bishop, 1988; Negrao et al., 1993) and inhibition of the nucleus tractus solitarius (Mueller and Hasser, 2006). By performing microinjections directly into the RVLM, our experiments provide evidence consistent with changes occurring at the level of the RVLM. Taken together, evidence from our study and others (Smith and Barron, 1990; Huber and Schreihofer, 2011) indicate that altered GABAergic modulation at the level of the RVLM plays an important role in changes in the regulation of blood pressure under physiological and pathophysiological states.

The observation of enhanced pressor responses to glutamate after blockade of GABA_A_ receptors also supports others' and our previous contention that sedentary conditions alter excitatory as well as inhibitory mechanisms at the level of the RVLM (Becker et al., 2005; Martins-Pinge et al., 2005; Mueller, 2010). Indeed, greater blood pressure or sympathoexcitatory responses to microinjections of glutamate in the RVLM have been observed in sedentary compared to physically active animals (Martins-Pinge et al., 2005; Mueller, 2007; Mischel and Mueller, 2011). The fact that responses to other excitatory neurotransmitters, such as angiotensin II, are not enhanced in sedentary animals (Becker et al., 2005) suggests that alterations in excitatory neurotransmission may be more specific to glutamatergic mechanisms; however, this remains to be fully established. The functional consequence of greater glutamatergic excitation of the RVLM may be reflected in an increased activation of the RVLM under various sympathoexcitatory conditions. For example, previous studies have demonstrated a greater number of Fos activated RVLM neurons in sedentary versus physically active animals following acute exercise (Ichiyama et al., 2002) and acute stress (Greenwood et al., 2003). As mentioned above, by activating the RVLM directly with microinjections of glutamate our studies corroborate enhanced activation at the level of the RVLM observed in previous studies. In combination, these results and ours suggest important alterations at the level of the RVLM that contribute to differences between sedentary and physically active animals.

Since lumbar sympathetic nerve responses to glutamate were equally enhanced in both groups by pretreatment with bicuculline, these data imply that lumbar sympathetic nerves are not responsible for the enhanced pressor responses observed in sedentary animals. One of the more straightforward explanations is that activation of other sympathetic nerves during glutamate microinjections in the RVLM could contribute to the enhanced pressor responses in sedentary animals. Indeed, several different sympathetic nerves can be activated by stimulation of the RVLM (Adams et al., 2007; Mueller et al., 2011 for example). Further studies involving a broader survey of sympathetic nerves would be required to confirm which sympathetic nerve(s) contribute to the enhanced pressor responses.

It is also possible that enhanced blood pressure responses in sedentary animals could be related to other pressor-related mechanisms produced by activation of the RVLM. For instance, we have previously shown that increases in blood pressure produced by intravenous administration of the α_1_-receptor agonist phenylephrine are enhanced in sedentary animals, suggesting some level of enhanced peripheral vasoconstriction (Mischel and Mueller, 2011). Since we did not observe a concentration-dependent enhancement of the pressor response under intact conditions even at the highest concentration of glutamate used (100 mM) we speculate that the greater pressor response is more likely due to enhanced activation of neurons controlling non-lumbar-mediated sympathetic nerve activity. Our studies emphasize the need to consider the highly integrative nature of centrally mediated changes in blood pressure and the multiple factors that could contribute to differences observed in various models (Osborn and Fink, 2010; Stocker et al., 2010; Mischel and Mueller, 2011; Osborn et al., 2011; Silva and Schreihofer, 2011; Osborn and Kuroki, 2012).

The lack of enhanced lumbar sympathetic nerve responses to activation of the RVLM in sedentary animals is in contrast to our previous study in which lumbar sympathetic nerve responses were significantly augmented in sedentary rats when compared to animals that were endurance trained on a treadmill (Mueller, 2007). It has been reported that rats allowed to run spontaneously on running wheels exhibit increases in maximal oxygen consumption, increases in heart weight-to-body weight ratio, and other exercise-related adaptations (Nelson et al., 2010). However, direct comparisons between treadmill and spontaneous wheel running studies should be done carefully given the number of differences between the models (e.g., voluntary vs. forced running; intensity, duration, length, and number of exercise bouts). Although animals in the current study ran over two kilometers per day on average and had lower body weights, these data alone do not imply that that these animals performed an equivalent type of exercise or have the same exercise capacity compared to treadmill trained animals in previous studies. Ultimately, these data highlight the importance of examining sympathetic nerve activity in the context of the different models of physical activity and inactivity.

To our knowledge there are only two other animal studies that have specifically examined the effects of chronic exercise on regulation of LSNA. Chen and colleagues reported modest changes in baroreflex control of LSNA in sedentary versus spontaneous wheel running rats (Chen and DiCarlo, 1996). Burgi and coworkers recently reported that resting lumbar nerve activity is reduced in treadmill trained versus sedentary Wistar–Kyoto rats (Burgi et al., 2011). In humans, the effects of exercise and inactivity on muscle sympathetic nerve activity appear to be fairly equivocal in terms of both resting (Ray and Hume, 1998) and reflex-mediated changes (Shoemaker et al., 1999; Fadel et al., 2001; Alvarez et al., 2005). Again these studies demonstrate the need for additional studies on the mechanisms by which different types of physical activity and inactivity influence regulation of sympathetic nerve activity to skeletal muscle.

### Technical considerations

Although previous studies have reported significant differences in baseline sympathetic nerve activity in various models of cardiovascular disease (Huber and Schreihofer, 2011; Mischel and Mueller, 2011; Silva and Schreihofer, 2011), comparisons of absolute levels of sympathetic nerve activity across different groups of animals warrants careful consideration (Guild et al., 2010; Burke et al., 2011). Since there were no differences in LSNA voltages between groups, we chose to express our data in terms of percent change to allow for an easier interpretation of the relative changes in sympathetic nerve activity. Furthermore, with our repetitive microinjection protocols we were able to examine nerve activity responses within animals over a time course that also demonstrated recovery of responses back to control values. Understanding that several important factors contribute to the voltage signal in a given recording (Guild et al., 2010; Burke et al., 2011) and group differences in the present study weren't significant, we have presented our nerve activity data in the more traditional and more easily interpreted format of percent change.

In order to complete our concentration response and repetitive microinjection protocols in the RVLM of rats while recording MAP, HR, and LSNA, we felt the use of anesthesia was necessary in these studies. The use of anesthesia allowed us to localize our RVLM microinjections more readily and successfully in a given animal since we were able to adjust the location of injection when necessary to achieve responses typically elicited from the RVLM. Furthermore, the use of triple-barrel glass micropipettes allowed us to inject different concentrations of glutamate or inject bicuculline or aCSF before and after multiple injections of glutamate without having to remove the micropipette from its position in the RVLM. Notwithstanding these advantages, we are fully aware that our use of anesthesia may have altered responses compared to those observed in conscious animals. Since we have been able to identify significant differences in our other studies using nearly identical preparations (Mueller and Hasser, 2006; Mueller, 2007; Mischel and Mueller, 2011), we are confident in the relative similarities and differences observed in the present study.

Given the time-consuming nature of our microinjections studies and the practicalities of generating the number of animals required for this study, we grouped experiments from animals that ran between 8 and 12 weeks. This can be considered a limitation of our study since it did not allow us to distinguish between potential differences between separate groups of animals that ran for 8 vs. 12 weeks. The time course by which functional alterations occur in brain regions important in cardiovascular regulation in response to physical (in)activity is worthy of consideration for future studies.

One caveat to our studies is that we used a volume and concentration of bicuculline based on previously published studies (Miyawaki et al., 2002; Moffitt et al., 2002; Horiuchi et al., 2004), including a study from our own laboratory in which we rigorously tested the effectiveness of GABA_A_ receptor blockade in the RVLM of physically active and sedentary rats (Mueller, 2007). Our expectation was that a similar volume (60 nl) and identical concentration (5 mM) of bicuculline used unilaterally would produce a similar level of blockade as to that used bilaterally in these previous studies. This line of thinking was reinforced by our experimental design in which we tested a higher volume of bicuculline (60 nl) against a smaller volume of glutamate (30 nl), both in the same micropipette, in order to maximize the chances that glutamate was only activating neurons in a region that had been affected by the antagonist. Nonetheless, the fact is that we did not perform experiments in the current study to confirm whether we achieved similar levels of blockade in both groups of animals. Consequently we cannot eliminate the possibility that GABA_A_ receptors were blocked to a lesser extent in the wheel running group and provide an explanation for the reduced effects of bicuculline on the blood pressure response to glutamate. We contend, however, that it is hard to reconcile the collective results of the current study with this possibility. For example, bicuculline produced similar effects on baseline MAP and SNA in both groups while producing differential effects on the glutamate-induced pressor response. Bicuculline also similarly enhanced the LSNA responses in both groups in a time course consistent with the known actions of bicuculline observed in previous studies (Miyawaki et al., 2002; Moffitt et al., 2002; Horiuchi et al., 2004; Mueller, 2007). Thus, it seems hard to conclude that these different effects could all occur because bicuculline produced less effective blockade in the wheel running group.

## Summary

The results of this study are reflective of others which have implicated important neural mechanisms in cardiovascular disease states (Esler et al., 2001; Schlaich et al., 2004; Guyenet, 2006; Fisher et al., 2009). Indeed, altered regulation of SNS activity from brainstem and hypothalamic cardiovascular nuclei have been demonstrated in several animal models of cardiovascular disease that are sensitive to physical activity or inactivity (Moffitt et al., 2002; Mueller, 2010; Patel and Zheng, 2012). Interestingly, altered glutamatergic or GABAergic signaling in the RVLM appears to be common to many of these disease states (Moffitt et al., 2002; Sved et al., 2003; Wang et al., 2009; Mueller, 2010; Huber and Schreihofer, 2011). To our knowledge, this is the first study to demonstrate selectively enhanced blood pressure responses to activation of the RVLM following blockade of tonic GABAergic inhibition in sedentary versus physically active animals. These data suggest that both glutamatergic and GABAergic regulation of RVLM neurons involved in blood pressure regulation are altered under different physical activity conditions and we speculate that these may play important roles in the development of cardiovascular diseases that are more prevalent in sedentary individuals (Blair, 2009; Danaei et al., 2009). In addition, since elevated sympathetic activity has detrimental effects on the cardiovascular system via direct and indirect mechanisms (Fisher et al., 2009; Grassi et al., 2011), this study and others highlight the need for more effective therapies which can lower sympathetic output specifically from the CNS (Mueller, 2010; Grassi et al., 2011; Osborn et al., 2011; Osborn and Kuroki, 2012).

### Conflict of interest statement

The authors declare that the research was conducted in the absence of any commercial or financial relationships that could be construed as a potential conflict of interest.
